# Comment on Fust et al. The Potential Economic Impact of the Updated COVID-19 mRNA Fall 2023 Vaccines in Japan. *Vaccines* 2024, *12*, 434

**DOI:** 10.3390/vaccines12101174

**Published:** 2024-10-17

**Authors:** Hannah R. Volkman, Jennifer L. Nguyen, Mustapha M. Mustapha, Luis Jodar, John M. McLaughlin

**Affiliations:** Pfizer Inc., New York, NY 10001, USA

**Keywords:** coronavirus, SARS-CoV-2, Japan, COVID-19, vaccination, cost-effectiveness

We noted three key inconsistencies in the Moderna-funded cost-effectiveness analysis by Fust et al. that compared the clinical and economic impact of using mRNA-1273 versus BNT162b2 in Japan’s 2023–2024 fall COVID-19 vaccination campaign published in *Vaccines* [1].

First, all modeled differences in clinical and economic outcomes between the two mRNA vaccines were based on the assumption that the vaccine effectiveness (VE) of mRNA-1273, which was assumed to be 84.9% (95% confidence interval [CI]: 65.7 to 93.3%) against hospitalization and 54.7% (40.3 to 65.6%) against infection, was meaningfully different compared to that of BNT162b2, which had an assumed effectiveness of 83.3% (81.9 to 84.5%) against hospitalization and 52.3% (51.3 to 53.2%) against infection. Notably, this assumed absolute difference in VE between the two mRNA COVID-19 vaccines is a mere two percentage points with clearly overlapping CIs for both endpoints. In real-world research, small differences in absolute VE like this are impossible to tease apart due to potential unmeasured biases and confounding present in observational studies.

The authors did not report 95% CIs nor any sensitivity analyses for the comparison between BNT162b2 and mRNA-1273 in Table 6 (as was done for mRNA-1273 versus no vaccine in Supplemental Table S4), which are needed to meaningfully interpret the accuracy and precision (or lack thereof) of model outcomes. Based on the small absolute differences in VE and overlapping 95% CIs used in the model, sensitivity analyses—had they been conducted—would likely have shown no clear difference between the two vaccines. Fust et al.’s suggestion that there were meaningful clinical differences between the two vaccines based on such small absolute differences in VE (with overlapping CIs) is akin to suggesting that BNT162b2 was more efficacious than mRNA-1273 at the initial time of licensure based on the small absolute differences in observed efficacy of the two vaccines (95.0% [90.3 to 97.6%] versus 94.1% [89.3 to 96.8%], respectively) [2,3]. Clearly this is flawed logic. Indeed, multiple studies have compared the booster dose VE of the two mRNA COVID-19 vaccines head-to-head during the Omicron era and found no significant difference [4,5,6,7]; however, none of these studies were cited by Fust et al. in their summary of the available evidence on the comparative VE of mRNA COVID-19 vaccines.

Second, how these small assumed absolute differences in VE between the two vaccines (i.e., 2.4% for infection and 1.6% for hospitalization with fully overlapping 95% CIs) somehow translated into considerably larger differences in terms of the total numbers of infections (4%) and hospitalizations (6%) averted when comparing the two vaccines in Table 6 is unclear.

Third and finally, it is curious that the authors were inconsistent in their approach to considering benefits and risks between BNT162b2 and mRNA-1273. Namely, when considering adverse events in Table 4, the authors assumed that the two vaccines had the same event rates based on overlapping 95% CIs. Yet, when it came to VE, the authors ignored the clearly overlapping 95% CIs in Table 1 and instead assumed a significant difference in the benefits of the vaccines.

The issues we have described above apply as well to other published articles by the authors that have compared the potential clinical and economic impact of BNT162b2 and mRNA-1273 vaccination programs in France [8], Germany [9], and the United States [10]; thus, the considerations we raise here have global implications beyond this individual analysis.

Currently, four years out from the start of the pandemic, the relevant public health question is not how effective mRNA COVID-19 vaccines are—as their effectiveness against COVID-19 has been well established over the last four years. Instead, it is why the uptake of these potentially life-saving vaccines has become so woefully low [11,12,13,14], even among those still at high risk of severe disease. Articles like this one from Fust et al. sadly distract public health officials from this more pertinent and pressing issue.

## Data Availability

Data availability is not applicable as no datasets were generated or analyzed during the generation of this Comment.

## References

[1] Fust K., Joshi K., Beck E., Maschio M., Kohli M., Lee A., Hagiwara Y., Van de Velde N., Igarashi A. (2024). The Potential Economic Impact of the Updated COVID-19 mRNA Fall 2023 Vaccines in Japan. Vaccines.

[2] Polack F.P., Thomas S.J., Kitchin N., Absalon J., Gurtman A., Lockhart S., Perez J.L., Pérez Marc G., Moreira E.D., Zerbini C. (2020). Safety and Efficacy of the BNT162b2 mRNA Covid-19 Vaccine. N. Engl. J. Med..

[3] Baden L.R., El Sahly H.M., Essink B., Kotloff K., Frey S., Novak R., Diemert D., Spector S.A., Rouphael N., Creech C.B. (2021). Efficacy and Safety of the mRNA-1273 SARS-CoV-2 Vaccine. N. Engl. J. Med..

[4] Liu B., Stepien S., Qian J., Gidding H., Nicolopoulos K., Amin J., Cheng A., Macartney K. (2023). Comparative effectiveness of four COVID-19 vaccines, BNT162b2 mRNA, mRNA-1273, ChAdOx1 nCov-19 and NVX-CoV2373 against SARS-CoV-2 B.1.1.529 (Omicron) infection. Vaccine.

[5] Gálvez J.M., Pinzón-Rondón Á.M., Chaparro-Solano H.M., Tovar-Romero H.V., Ramírez-Prieto J., Ortigoza-Espitia S.A., Ruiz-Sternberg Á.M. (2023). Effectiveness of the Booster Dose in Protecting against COVID-19, Colombia 2022. Vaccines.

[6] Chin E.T., Leidner D., Lamson L., Lucas K., Studdert D.M., Goldhaber-Fiebert J.D., Andrews J.R., Salomon J.A. (2022). Protection against Omicron from Vaccination and Previous Infection in a Prison System. N. Engl. J. Med..

[7] Kirk B., Bush C., Toyip A., Mues K.E., Beck E., Li L., St. Laurent S., Georgieva M., Marks M.A., Sun T. (2023). Real-world comparative effectiveness of a third dose of mRNA-1273 versus BNT162b2 among adults aged ≥65 years in the United States. medRxiv (Pre-Print).

[8] Lee A., Davido B., Beck E., Demont C., Joshi K., Kohli M., Maschio M., Uhart M., El Mouaddin N. (2024). Substantial reduction in the clinical and economic burden of disease following variant-adapted mRNA COVID-19 vaccines in immunocompromised patients in France. medRxiv (Pre-Print).

[9] Joshi K., Scholz S., Maschio M., Kohli M., Lee A., Fust K., Ultsch B., Van de Velde N., Beck E. (2024). Clinical impact and cost-effectiveness of the updated COVID-19 mRNA Autumn 2023 vaccines in Germany. J. Med. Econ..

[10] Kohli M.A., Maschio M., Joshi K., Lee A., Fust K., Beck E., Van de Velde N., Weinstein M.C. (2023). The potential clinical impact and cost-effectiveness of the updated COVID-19 mRNA fall 2023 vaccines in the United States. J. Med. Econ..

[11] Centers for Disease Control and Prevention COVID-19 Vaccine Uptake and CDC’s Commitment to Vaccine Equity 2023. https://www.cdc.gov/ncird/whats-new/vaccine-equity.html.

[12] Centers for Disease Control and Prevention Updated 2023–24 COVID-19 Vaccination Coverage, Adults 65 Years and Older, United States 2024. https://www.cdc.gov/covidvaxview/weekly-dashboard/adults-65yrs-older-vaccination.html?CDC_AAref_Val=https://www.cdc.gov/vaccines/imz-managers/coverage/covidvaxview/interactive/adults-65yrs-over-coverage.html.

[13] European Centre for Disease Prevention and Control COVID-19 Vaccine Tracker 2023. https://vaccinetracker.ecdc.europa.eu/public/extensions/COVID-19/vaccine-tracker.html#uptake-tab.

[14] Prime Minister’s Office of Japan COVID-19 Vaccines—Total Number of Vaccines Since Autumn 2023 2024. https://japan.kantei.go.jp/ongoingtopics/vaccine.html.

